# Physical activity, physical frailty and depressive symptoms among Chinese male and female older adults: do different indicators make a difference?

**DOI:** 10.3389/fpubh.2023.1280952

**Published:** 2023-11-27

**Authors:** Ying Wang, Xue Wang, Xinyu Zhu, Yawen Sun, Buxin Han, Tianyong Chen

**Affiliations:** ^1^CAS Key Laboratory of Mental Health, Institute of Psychology, Beijing, China; ^2^Department of Psychology, University of Chinese Academy of Sciences, Beijing, China; ^3^Department of Rehabilitation, General Hospital of Tianjin Medical University, Tianjin, China

**Keywords:** physical activity, physical frailty, grip strength, depressive symptoms, older adults

## Abstract

**Objectives:**

Older adults become more inactive and frailer with aging. Physical status is closely linked to mental health, but it is unclear which physical indicator is more strongly associated with depressive symptoms in older adults. The present study aimed to compare relationships between self-reported physical activity, physical frailty (muscle mass, muscle strength, and gait ability) and depressive symptoms in community male and female older adults.

**Methods:**

A total of 1,180 adults aged 60 years and older were recruited to participate in this study from a Chinese community receiving annual check-up service from September 2018 to May 2019. Physical activity was assessed by the International Physical Activity Questionnaire (IPAQ). The Bio-electrical Impedance Analyzer was used to determine the muscle mass. As the indicators of muscle function, grip strength and gait ability were assessed by the dynamometer and Timed Up and Go Test (TUGT), respectively. The 15-item version of Geriatric Depression Scale (GDS-15) was used to examine depressive symptoms. Demographic variables, health status and sleep quality were collected using questionnaire.

**Results:**

11.8% men and 11.9% women reported depressive symptoms. Logistic regression showed that depressive symptoms was associated with low grip strength (*OR* = 2.42, 95% *CI*: 1.04–5.63), slow gait ability (*OR* = 3.60, 95% *CI*: 1.28–10.13) in older males, and associated with low level of self-reported physical activity (*OR* = 3.85, 95% *CI*: 2.00–7.42) in older females. No significant association was found between muscle mass and depressive symptoms.

**Conclusion:**

There were gender differences in the relationship between physical activity, physical frailty, and depressive symptoms. Grip strength and gait ability may be a better indicator of frailty for predicting depressive symptoms in older men while physical activity may be useful in predicting depressive symptoms in older women.

## 1 Introduction

It is well established that the health of body and mind are inextricably linked (1, 2). Older adults become more inactive and frailer with aging, and their psychological problems become more prominent (3). Previous studies have explored the relationship between physical status and depressive symptoms in older adults, while there are still some disagreements in the results using different indicators.

Mental health benefits conferred by physical activity are wildly recognized (4). In cross-sectional studies, researchers have found that insufficient physical activity predispose older adults to depressive symptoms and vice versa (5, 6). Longitudinal study also provided increasing clarity on the benefits of physical activity, showed that the odds of incident depression were 23% lower by meeting physical activity guidelines (7). Only a few previous studies failed to find the benefits of physical activity, and this may come from improper evaluation tools which easily exaggerate the amount of exercise (8). However, based on the gender differences in biological structure and function and in physical activity preference and attraction across the lifetime, some scholars began to pay attention to the gender difference in health benefits derived from physical activity (9). For example, it was found that the reduction of vigorous physical activity was associated with increased odds for depressive symptoms and higher level of recreational physical activity was associated with less depressive symptoms among older females, while there was no such correlation in men (10, 11).

More and more studies have linked physical frailty to higher risk of depressive symptoms in older adults (12, 13) as well, while associations between frailty using different indicators (such as muscle strength, gait ability and muscle mass) and depressive symptoms remain inconsistent. Brooks and colleagues found that grip strength was negatively associated with depressive symptoms among older adults in the American community (14). A study of six low-and-middle-income countries also suggested that adults over 50-year-old with lower grip strength suffer a higher prevalence of depressive symptoms (15). Both cross-sectional and longitudinal studies support this relationship in Japan (16). In Norway, it was found that older adults with lower gait ability suffered more depressive symptoms (17). In brief, majority of the literature support relationships between grip strength, gait ability and depressive symptoms, where grip strength and gait ability represent upper and lower physical capacity respectively. However, inconsistencies between studies raise concerns about whether there is a correlation between muscle mass and depressive symptoms. Wu et al. (18) found that both muscle mass and muscle strength were negatively correlated with depressive symptoms, the result of a national Korean study suggested no correlation between muscle mass and depressive symptoms in any age group (19). Moreover, there are great differences in muscle strength and muscle mass between men and women. The physiological difference may lead to a gender difference between frailty and depressive symptoms (20). Therefore, the predictive effect of physical activity and physical frailty on depressive symptoms in the older adults of different gender remains to be examined further.

In summary, the objectives of the present study were to: (i) investigate the association between physical activity, different indicators of frailty (muscle strength, gait ability and muscle mass) and geriatric depressive symptoms; and (ii) explore the possible gender differences in these relationships.

## 2 Methods

### 2.1 Participants

The participants in this study were community-dwelling older adults age 60 years or more. Between September 2018 and May 2019, a total of 1,180 sixty-year-olds were recruited from the community older adults receiving annual check-up service voluntarily at the Community Healthcare Center of Nanyingmen street, Heping District, Tianjin. Because blood samples were collected, the participants were instructed not to eat or drink anything before the examination. Exclusion criteria for participation included a history of major depression, unable to communicate, unable to complete grip strength or walking test (e.g., severe arthritis or surgery). A total of 1,180 older adults have participated in this study. Since there were 55 men and 80 women who did not have the body composition test, we interpolated the missing value using mean values of different genders and different age stages of the appendicular skeletal muscle mass. The study was approved by the Ethics Committee of the Institute of Psychology of the Chinese Academy of Sciences (IPCAS). All participants signed an informed consent.

### 2.2 Measures

#### 2.2.1 Depressive symptoms

The 15-item version of Geriatric Depression Scale (GDS-15) was administered to all participants (21). A validation study by Boey (22) supported the use of GDS-15 as a reliable measurement utilized among older adults in urban China. All items in the GDS-15 are rated by self-report and scores are summed, resulting in a possible total score of 0–15. We defined depressive symptoms as a score of 5 or more (23).

#### 2.2.2 Physical activity

Physical activity was assessed using the Chinese version of International Physical Activity Questionnaire (IPAQ), a self-report instrument that assesses physical activity during the past seven days (24). A total physical activity score was calculated as the metabolic equivalent of energy per week (MET/week). MET per week for each participant were calculated as follows: Total activity (MET/ week) = (3.3 × walking minutes per day × walking days) + (4.0 × moderate activity minutes per day × moderate activity days) + (8.0 × vigorous activity minutes per day × vigorous activity days). There were high reliability and validity in IPAQ for Chinese population (25). A total score below 600 MET-min/w was coded as “low level of physical activity,” 600 to 3000 MET-min/w was coded as “moderate level of physical activity,” and above 3000 MET-min/w was coded as “high level of physical activity” (26).

#### 2.2.3 Physical frailty

Physical frailty was indicated by muscle strength, muscle mass and gait ability. Muscle strength of the dominant hand was performed with a hand-held dynamo-meter (GRIP-D; Takei, Niigata, Japan), since grip strength is closely related to total body strength and widely used to evaluate the muscle strength in older participants (27). The highest value from 3 trials in a standing position was used in the analysis.

The calculation of the muscle mass index was performed by dividing the appendicular skeletal muscle mass (ASM) by the body weight (ASM/ wt), differences related to height, sex, age and race were eliminated when using body weight as the denominator (28). The Bio-electrical Impedance Analyzer (INBody 720; Biospace Co., Ltd, Seoul, Korea) was used to determine the ASM, while participants standing on 2 metallic electrodes and held metallic grip electrodes. Muscle strength and muscle mass have been distributed according to quartile and conducted a sex-stratified analysis for the large gender gap.

Measurement of gait ability was performed by the Time Up and Go Test (TUGT) (29). It measures the time taken by a 6-meter walk, which requires participants to rise from a standard chair (with the height of 45 cm), walk three meters, turn around, walk back to the chair, and sit down without any device throughout.

#### 2.2.4 Control variables

Demographic information (marital status, sex, age and education), health (obesity and disease history) and sleep quality were assessed using questionnaire items. Body mass index (BMI), calculated as weight (kg)/height squared (m^2^), was used to indicate obesity (BMI ≥ 28: obesity = 1; BMI < 28: obesity = 0) (30). Sleep quality was assessed by one item and coded as 3 levels (1–3 were good, fair and poor).

### 2.3 Data analysis

All analyses were conducted using SPSS V.24.0, stratified by gender. Frequencies and percentages were computed for each demographic characteristic. Bivariate analyses with Chi-square test were used to explore the relevance of demographic variables, physical activity, physical frailty to depressive symptoms.

After adjusting for age, marital status, education, disease, sleep quality and obesity, logistic regression analysis was used to test relationships between indicators of physical status and depressive symptoms. Grip strength, gait ability, muscle mass and physical activity was added in model 1, model 2, model 3, and model 4, respectively. Afterwards, we added indicators of physical activity and physical frailty to the same model to test the independent association between each variable and depressive symptoms.

## 3 Results

The characteristics and demographics of the participants are shown in Table 1. The analysis include data from 1,180 older adults, of whom 533 were men (mean age: 68.07 ± 6.47) and 647 were women (mean age: 66.52 ± 5.19). In addition, it is consistent with the basic situation of old adults in China that women have lower levels of education, higher rates of widowhood and obesity. Of all the participants, 11.9% were classified as being depressive symptoms. The proportions of depressive symptoms in men and women were 11.8 and 11.9% respectively, with no significant difference (χ^2^ = 0.01, P = 0.966).

**Table 1:** Characteristics of the study population.

	**Men (*n =* 533)**	**Women (*n =* 647)**
**Variable**	***N*** **(%)**	***N*** **(%)**
**Age (years)**		
60–65	193 (36.2)	280 (43.3)
65–69	155 (29.1)	205 (31.7)
≥70	185 (34.7)	162 (25.0)
**Marital status**		
Married	475 (89.1)	522 (80.7)
Not married	58 (10.9)	125 (19.3)
**Education**		
Illiteracy	93 (17.4)	205 (31.7)
Elementary school	276 (51.8)	327 (50.5)
Middle school and above	164 (30.8)	115 (17.8)
**Obesity**		
No	435 (81.6)	482 (74.5)
Yes	98 (18.4)	165 (25.5)
**ASM/wt (%)**		
Level 1	133 (25.0)	162 (25.0)
Level 2	131 (24.6)	160 (24.7)
Level 3	136 (25.5)	163 (25.2)
Level 4	133 (25.0)	162 (25.0)
**Grip strength (kg)**		
Level 1	137 (25.7)	168 (26.0)
Level 2	133 (25.0)	157 (24.3)
Level 3	132 (24.8)	161 (24.9)
Level 4	131 (24.6)	161 (24.9)
**Gait ability (s)**		
0–9	310 (58.2)	316 (48.8)
10–14	193 (36.2)	298 (46.1)
≥15	30 (5.6)	33 (5.1)
**Physical activity**		
Low	73 (13.7)	156 (24.1)
Moderate	241 (45.2)	283 (43.7)
High	219 (41.1)	208 (32.1)
**Sleep quality**		
Good	273 (51.2)	267 (41.3)
Fair	211 (39.6)	270 (41.7)
Poor	49 (9.2)	110 (17.0)
**Disease**		
No	272 (51.0)	228 (35.2)
Yes	261 (49.0)	419 (64.8)

ASM/wt, appendicular skeletal mass adjusted by body weight. The percentage for each variable (more than two categories) should add up to 100, but may not due to rounding errors.

Chi-square tests were conducted to analysis associations between depressive symptoms and other variables. As shown in Table 2, a significant higher prevalence of depressive symptoms was observed in men with old age, no spouse, low grip strength, low gait ability and poor sleep quality (*P* < 0.05) and in women with old age, disease history, low grip strength, low physical activity and no spouse (*P* < 0.05).

**Table 2:** Bivariate analyses of association between depressive symptoms and related factors.

**Variable**	**Men (*****n** = * **533)**			**Women (*****n** = * **647)**		
	**Cases (%)**	*χ^2^*	***P***	**Cases (%)**	*χ^2^*	***P***
**Age (years)**		6.26	0.044		8.01	0.018
60–65	14 (7.3)			25 (8.9)		
65–69	21 (13.5)			23 (11.2)		
≥70	28 (15.1)			29 (17.9)		
**Marital status**		4.91	0.027		9.69	0.002
Married	51 (10.7)			52 (10.0)		
Not married	12 (20.7)			25 (20.0)		
**Education**		4.73	0.094		2.46	0.292
Illiteracy	5 (5.4)			28 (13.7)		
Elementary school	38 (13.8)			40 (12.2)		
Middle school and above	20 (12.2)			9 (7.8)		
**Obesity**		2.34	0.126		0.01	0.919
No	47 (10.8)			57 (11.8)		
Yes	16 (16.3)			20 (12.1)		
**ASM/wt (%)**		0.35	0.95		1.09	0.78
Level 1	17 (12.8)			19 (11.7)		
Level 2	16 (12.2)			20 (12.5)		
Level 3	16 (11.8)			22 (13.5)		
Level 4	14 (10.5)			16 (9.9)		
**Grip strength (kg)**		12.83	0.005		8.60	0.035
Level 1	27 (19.7)			21 (12.5)		
Level 2	16 (12.0)			28 (17.8)		
Level 3	9 (6.8)			15 (9.3)		
Level 4	11 (8.4)			13 (8.1)		
**Gait ability (s)**		7.91	0.019		4.39	0.112
0–9	30 (9.7)			29 (9.2)		
10–14	25 (13.0)			43 (14.4)		
≥15	8 (26.7)			5 (15.2)		
**Physical activity**		3.26	0.196		30.61	< 0.001
Low	12 (16.4)			38 (24.4)		
Moderate	31 (12.9)			24 (8.5)		
High	20 (9.1)			15 (7.2)		
**Sleep quality**		7.99	0.018		5.78	0.056
Good	22 (8.1)			25 (9.4)		
Fair	32 (15.2)			32 (11.9)		
Poor	9 (18.4)			20 (18.2)		
**Disease**		0.00	0.968		5.39	0.020
No	32 (11.8)			18 (7.9)		
Yes	31 (11.9)			59 (14.1)		

ASM/wt, appendicular skeletal mass adjusted by body weight.

ORs from the logistic regression model for depressive symptoms according to physical frailty and physical activity are shown in Tables 3, 4. Results showed that some indicators of physical frailty rather than physical activity have been linked to depressive symptoms in older males. Male participants with lower grip strength (*OR* = 2.42, 95% *CI*: 1.04–5.63) and lower gait ability (*OR* = 3.6, 95% *CI*: 1.28–10.13) had significant higher OR for depressive symptoms, but the role of muscle mass remains unclear. In addition, obesity and poor sleep quality were found to be a risk factor of depressive symptoms from all four models (Model 1: *OR* = 2.27, 95% *CI*: 1.14–4.51, *P* = 0.020; Model 1: *OR* = 3.53, 95% *CI*: 1.44–8.68, *P* = 0.006).

**Table 3:** Logistic regressions of physical activity, frailty on depressive symptoms in men.

**Variable**	**B**	**SE**	**Wald**	**OR (95% CI)**	**P**
**Model 1**					
Grip strength (Ref: ≥39.2 kg)			8.69		0.034
0–29.0	0.88	0.43	4.20	2.42 (1.04, 5.63)	0.040
29.1–33.9	0.36	0.44	0.66	1.43 (0.60, 3.40)	0.416
34.0–39.1	−0.30	0.49	0.39	0.74 (0.28, 1.92)	0.533
**Model 2**					
Gait ability (Ref: 0–9s)			5.93		0.051
10–14s	0.33	0.32	1.08	1.39 (0.75, 2.59)	0.298
≥15s	1.28	0.53		3.60 (1.28, 10.13)	0.015
**Model 3**					
ASM/wt (Ref: ≥30.14)			0.14		0.986
0–21.30	0.05	0.42	0.02	1.05 (0.46, 2.39)	0.902
21.31–26.06	0.07	0.40	0.03	1.07 (0.49, 2.35)	0.872
26.07–30.13	−0.07	0.41	0.03	0.94 (0.42, 2.07)	0.868
**Model 4**					
PA (Ref: High)			3.56		0.169
Low	0.75	0.42	3.27	2.12 (0.94, 4.80)	0.071
Moderate	0.42	0.32	1.74	1.52 (0.82, 2.84)	0.187

ASM/wt, appendicular skeletal mass adjusted by body weight; PA, physical activity. Model 1–4, adjusted for age, marital status, education, disease, sleep quality and obesity.

**Table 4:** Logistic regressions of physical activity, frailty on depressive symptoms in women.

**Variable**	**B**	**SE**	**Wald**	**OR (95% CI)**	**P**
**Model 1**					
Grip strength (Ref: ≥24.2 kg)			4.68		0.197
0–17.4	0.16	0.41	0.16	1.18 (0.53, 2.61)	0.692
17.5–20.6	0.68	0.38	3.26	1.97 (0.94, 4.1)	0.071
20.7–24.1	0.14	0.40	0.13	1.15 (0.52, 2.54)	0.724
**Model 2**					
Gait ability (Ref: 0–9s)			1.09		0.581
10–14s	0.28	0.27	1.05	1.32 (0.78, 2.24)	0.305
≥15s	0.26	0.55	0.23	1.30 (0.44, 3.80)	0.633
**Model 3**					
ASM/wt (Ref: ≥33.52)			0.86		0.834
0–24.91	0.23	0.38	0.36	1.26 (0.59, 2.67)	0.552
24.92–29.52	0.30	0.37	0.65	1.35 (0.65, 2.79)	0.421
29.53–33.51	0.30	0.36	0.70	1.36 (0.67, 2.77)	0.403
**Model 4**					
PA (Ref: High)			25.72		< 0.001
Low	1.35	0.34	16.25	3.85 (2.00, 7.42)	< 0.001
Moderate	0.06	0.35	0.03	1.06 (0.53, 2.11)	0.873

ASM/wt, appendicular skeletal mass adjusted by body weight; PA, physical activity. Model 1–4, adjusted for age, marital status, education, disease, sleep quality and obesity.

For women group, no significant associations were found between muscle strength, muscle mass, gait ability and depressive symptoms. However, older females with low level of physical activity (<600 MET-min/w) were 3.85 times more likely to be depressed than that with high level of physical activity (more than 3000 MET-min/w). These effects still exist when we add physical activity and physical frailty in one model.

## 4 Discussion

This is a community-based cross-sectional study aimed to explore the relationship between physical activity, physical frailty and geriatric depressive symptoms. The major findings are that lower amount of physical activity is significantly associated with depressive symptoms for older females, and depressive symptoms in the older males was associated with lower grip strength and lower gait ability.

The benefit of physical activity on health is widely recognized. Previous studies have shown that physical activity in later life can extend lifespan and reduce the risk of disability (31). Our results are in line with those studies advocating that physical activity is a protective factor against depressive symptoms in older participants (32, 33). Neuromolecular mechanisims that physical activity and antidepressants shared have been proposed in the past decade, including neurotrophic factor expression (34, 35), HPA axis response (36–38), anti-inflammatory effects (39, 40).

However, this effect was only observed in female participants in this study. Although previous researchers may not pay enough attention to gender differences, several studies analyzed by gender stratification showed similar results (10, 11, 41–43). An earlier study from Nepal, by contrast, only found this effect in men. Nevertheless, the effect was modest and researchers only used simple questions to assess the frequency of activity, which may undermine the credibility (44). The gender difference may partly be explained by psychological mechanisms of physical activity on depressive symptoms, such as increasing self-esteem, self-efficacy, and social support (45–48). It was found that physical activity with social attributes help alleviate depressive symptoms (49), while physical activity related to work, transport or domestic activity was not associated with depressive symptoms (50). From this perspective, women have stronger social connections than men and may be able to amplify the benefits of physical activity (51). On the other hand, the brain benefits from exercise also differ between men and women, with studies showing that daily walking improves hippocampal volume in women, but not in men (52). Due to the close connection between depressive symptoms and hippocampus (53), this may also be a potential physiological mechanism for gender differences.

The association between physical frailty and depressive symptoms is consistent with the observations of Ji et al. (54) and Liu et al. (55), showed that older persons with frailty are tend to have depressive symptoms. Muscle is closely associated with physical frailty, which is also a secretory organ (56) and contributes benefits on mental health via secretion of numerous myokines (57) and neurotrophic factor (58). Decline of muscle mitochondrial function is associated with depression as well (59). Malnutrition (60), hormonal imbalances (61), cardiovascular disease and inflammation (62) may also play a role in this relationship.

Maintaining muscle mass, improving muscle strength and physical function are important for preventing frailty (63, 64). However, not every indicator was associated with depressive symptoms in this study, and there were gender differences in the results. Previous studies exploring the relationship between muscle mass and depressive symptoms have yielded inconsistent results. Two studies reported associations between muscle mass and depressive symptoms, with one of them only found this relationship in male participants (18, 65). Unlike these two, the present study did not show significant association between them, which is consistent with a national Korean study (19). Unlike muscle mass, the predictive effects of muscle strength and gait ability on depressive symptoms were consistent with previous studies (66–68). On the one hand, the degree of physical frailty among community older adults in the present study may not be sufficient to trigger higher rate of depressive symptoms or more severe depressive symptoms, which may weaken the relationship of body and mind. On the other hand, it is possible that muscle function, rather than muscle mass, may be more closely related to health outcomes. For example, Chen et al. (69) found that it may be muscle function rather than muscle mass contribute to depression development via malnutrition. Arts et al. (70) also suggested that only performance-based physical frailty (encompassing gait ability and handgrip strength) was associated with higher levels of inflammatory markers and hence depression. Similarly, Newman et al. (71) clarified that low grip strength rather than muscle mass is associated with total mortality rates in late life.

As to the gender difference, these results are contrary to a recent study among rural older South Africans (72), but similar to a meta-analysis that found grip strength was associated with a reduced risk of depressive symptoms in male but not in female (73). The possible reason for gender difference may be related to the loss of skeletal muscle strength, with male muscle strength decreases earlier and faster than female (74, 75). Future research is needed to explore this issue.

## 5 Implications

This study explored the associations between physical activity, physical frailty and depressive symptoms in older adults, and provided support for the relationship of body and mind. The pathophysiology pathway between physical status and depressive symptoms is worth exploring because of the gender differences. Future studies on the relationship between body and mind may consider the two variables and pay attention to the possible gender differences. Furthermore, they can be used as supplementary indicators to identify the risk of depression in older persons, and also as a potential target for intervention. It is hoped that this study will contribute to a deeper understanding for the prevention and early detection of depression.

## 6 Strengths and limitations

This study has some strengths. First, we collected data through an annual physical check-up, with large number of participants and good representativeness. Second, to the best of our knowledge, this is the first study to focus on different indicators of physical frailty and depressive symptoms from a gender perspective, which has helped us further understand the relationship between body and mind.

This study also has some limitations. First, this is a cross-sectional study, which is not sufficient to establish a causal relationship between physical activity, physical frailty and depressive symptoms. Longitudinal studies can be carried out in the future. Second, this study used a self-reported questionnaire to assess the physical activity of old adults in the past week. It is possible that a few participants were less involved than usual in the previous 7 days due to injury or other reasons, leading to a potential bias. Third, since this study is based on an annual physical check-up, we were not able to use objective measurements such as pedometers, which may reflect the physical activity of older adults more accurately.

## Data availability statement

The raw data supporting the conclusions of this article will be made available by the authors, without undue reservation.

## Ethics statement

The studies involving humans were approved by the Ethics Committee of the Institute of Psychology of the Chinese Academy of Sciences (IPCAS). The studies were conducted in accordance with the local legislation and institutional requirements. Written informed consent for participation was not required from the participants or the participants' legal guardians/next of kin in accordance with the national legislation and institutional requirements.

## Author contributions

YW: Validation, Writing – original draft, Writing – review & editing. XW: Data curation, Investigation, Methodology, Writing – original draft. XZ: Writing – review & editing, Writing – original draft. YS: Writing – review & editing. BH: Project administration, Supervision, Writing – review & editing. TC: Project administration, Supervision, Writing – review & editing.
